# Current News

**Published:** 1897-10

**Authors:** 


					﻿
Current News.
NATIONAL ASSOCIATION OF DENTAL EXAMINERS.
At the annual session of the National Association of Dental
Examiners, held at Old Point Comfort, Va., July 30 to August 2,
1897, twenty-two States present, the following officers were elected
for the new year:
President, C. G. Edwards, D.D.S., Louisville, Ky.; Vice-Presi-
dent, G. L, Parmele, D.M.D., Hartford, Conn.; Secretary and
Treasurer, Charles A. Meeker, D.D.S., Newark, N. J.
The president appointed as members of the Committee on
Colleges, G. Carleton Brown, D.D.S., chairman, Elizabeth, N. J.;
J. A. Hall, Collinsville, Ala.; M. H. Chippell, D.D.S., Knightstown,
Ind.; L. Ashley Faught, D.D.S., secretary by appointment of the
committee, 1415 Walnut Street, Philadelphia, Pa.
MASSACHUSETTS BOARD OF REGISTRATION IN DEN-
TISTRY.
A meeting of the Massachusetts Board of Registration in Den-
tistry will be held in Boston, Monday, November 15, 1897. Appli-
cation should be made at once to
E. V. McLeod, D.D.S.,
Secretary.
New Bedford, Mass.
PENNSYLVANIA EXAMINING BOARD.
Governor Hastings has appointed the following State Dental
Examining Board under the new law :
Harry Gerhart, Lewisburg, and Jesse C. Green, West Chester,
to serve one year; C. W. Klump, Williamsport, and J. A. Libby,
Pittsburg, to serve two years; C. V. Kratzer, Reading, and H. E.
Roberts, Philadelphia, to serve three years.
				

